# Expanding the boundaries of local similarity analysis

**DOI:** 10.1186/1471-2164-14-S1-S3

**Published:** 2013-01-21

**Authors:** W Evan Durno, Niels W Hanson, Kishori M Konwar, Steven J Hallam

**Affiliations:** 1 Department of Microbiology & Immunology, University of British Columbia, Vancouver, BC, Canada; 2 Graduate Program in Bioinformatics, University of British Columbia, Vancouver, BC, Canada

## Abstract

**Background:**

Pairwise comparison of time series data for both local and time-lagged relationships is a computationally challenging problem relevant to many fields of inquiry. The Local Similarity Analysis (LSA) statistic identifies the existence of local and lagged relationships, but determining significance through a *p*-value has been algorithmically cumbersome due to an intensive permutation test, shuffling rows and columns and repeatedly calculating the statistic. Furthermore, this *p*-value is calculated with the assumption of normality -- a statistical luxury dissociated from most real world datasets.

**Results:**

To improve the performance of LSA on big datasets, an asymptotic upper bound on the *p*-value calculation was derived without the assumption of normality. This change in the bound calculation markedly improved computational speed from *O*(*pm*^2^*n*) to *O*(*m*^2^*n*), where *p *is the number of permutations in a permutation test, *m *is the number of time series, and *n *is the length of each time series. The bounding process is implemented as a computationally efficient software package, FASTLSA, written in C and optimized for threading on multi-core computers, improving its practical computation time. We computationally compare our approach to previous implementations of LSA, demonstrate broad applicability by analyzing time series data from public health, microbial ecology, and social media, and visualize resulting networks using the Cytoscape software.

**Conclusions:**

The FASTLSA software package expands the boundaries of LSA allowing analysis on datasets with millions of co-varying time series. Mapping metadata onto force-directed graphs derived from FASTLSA allows investigators to view correlated cliques and explore previously unrecognized network relationships. The software is freely available for download at: http://www.cmde.science.ubc.ca/hallam/fastLSA/.

## Background

The exponential increase and ubiquitous use of computational technology has given rise to an era of "Big Data" that pushes the limits of conventional data analysis [1-3]. Techniques for analyzing big datasets often proceed by identifying patterns of co-occurrence or correlation through principal component analysis (PCA) [4], multidimensional scaling (MDS) [5], etc. However, many of these methods require significant data reduction or smoothing which makes them difficult to interpret [6]. Other methods such as multiple linear regression or Pearson's correlation coefficient (PCC) are easy to interpret as they operate on data in their native data space, without any kind of large data transformation or dimensionality reduction, but are limited in the structure that they can detect.

Though PCC is a classic and powerful technique for finding linear relationships between two variables, it is not designed for capturing lead-lag relationships seen in time series data. Local similarity analysis (LSA) [6] extends correlation calculations to include the time variable, enabling identification of local correlates. Furthermore, Ruan *et al. *have presented a graphical network framework in which to visualize the structure of significant LSA correlations. Unfortunately, the current implementation of LSA requires multiple runs on permuted data and a Monte Carlo statistical method known as a *permutation test *to evaluate a null distribution and obtain a *p*-value determining significance. Each iteration of this procedure has a computational complexity of *O*(*pm*^2^*n*), where *p *is the number of permutations, *m *is the number of covariate time series, and *n *is their length. Due to the number of pair-wise calculations needed, extant LSA is computationally onerous when *m *is large, limiting its use to datasets where the number of observed variables at each time point is small (< 100). Though there has been some improvement to its performance [7], assumptions of normality and implementation issues continue to stymie practical application of LSA on big datasets.

Here we describe a novel asymptotic upper bound on the calculation of the LSA statistic's *p*-value, resulting in an exponentially converging calculation to bound and check for significance of computed LSA statistics without a computationally intensive permutation test. This bound does not require a rank-vector normal transformation, promoting application to any distribution that has finite variance. As a result, this implementation of LSA can navigate big datasets with millions of co-variate time series. We demonstrate this using time-series datasets from public health [8], microbial ecology [9], and social media [10]. The implemented algorithm, named FASTLSA, is written in C and optimized for threading on multi-core computers.

### Interpreting the LSA statistic

**Figure 1:**
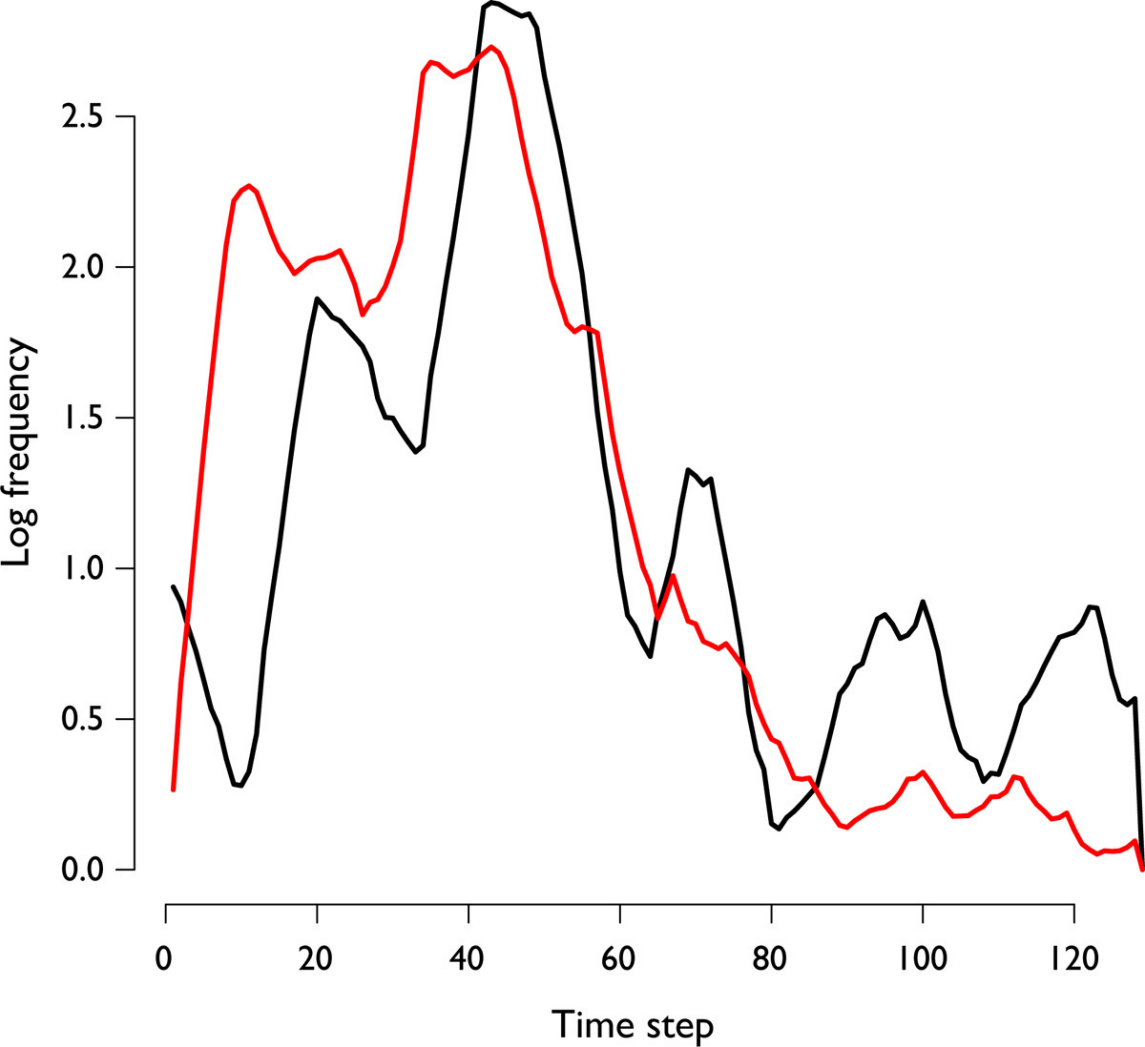
**A lagged correlation between two time series**. An example of two set time series that contain a lead-lag correlation.

LSA concerns itself with pairs of time series data. The LSA Statistic can be interpreted in a manner similar to PCC when no lag window exists between two time series. However, LSA is also capable of capturing localized correlation that is staggered or lagged. A large positive or negative LSA value indicates a correspondingly strong PCC correlation or a correlation at a time displacement within the lag window (Figure 1). Note that if both positive and negative correlations exist between two time series, LSA will only report the strongest of the two.

LSA is advantageous on large datasets containing many time series. Results can be visualized as a graphical network where nodes represent the individual time series and the edges represent their LSA correlation statistic. When displayed using a force-directed layout in Cytoscape [11], closely related time series cluster together, visually isolating clusters of local similarity. Metadata related to experimental or environmental conditions can then be applied to the nodes, shedding insight into hierarchical network structure.

## Implementation

### Description of the LSA algorithm

In this section we reproduce the algorithm from [6] to compute LSA statistics and their corresponding *p*-values between pairs of time series in a dataset. We assume as input a set of time series vectors of equal length. Let us denote the number of time series by *m *and their length as *n*. Let us denote the time series dataset as **X **where *X_ij _*denotes the *j*th element of the *i*th time series, with *i *= 1, 2, ..., *m *and *j *= 1, 2, ..., *n*, and assume that the *X_ij _*are real numbers. We also assume that there are no missing values in the dataset **X**, and realize that practical use will require interpolation or filtering.

**Figure 2:**
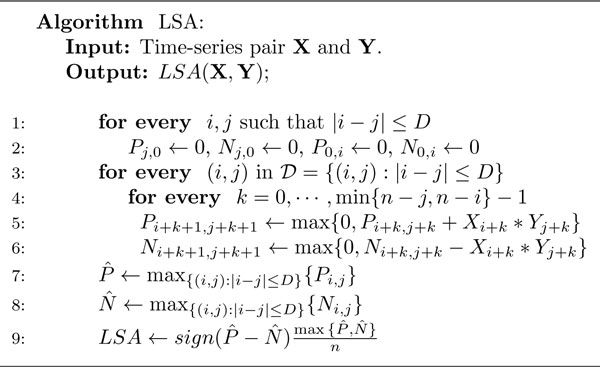
**The LSA algorithm**. Algorithm for computing the LSA for a pair of time series **X **and **Y**. D  denotes the set $\left\{\left(i,j\right):i,j\in {\mathbb{N}}^{+}\text{, either}\;i=\;0\text{ or}\;j=0\text{ and}\;\left|i-j\right|\leq D\right\}$ and ${\mathbb{N}}^{+}\;$ denotes the set of positive integers.

In Figure 2 we present the algorithm for computing the LSA statistic for a pair of time series, $X={\left\{X_{i}\right\}}_{1}^{n}$ and $Y={\left\{Y_{i}\right\}}_{1}^{n}$, where the length of the time series is assumed to be equally spaced in time. We have modified the presentation of the LSA algorithm by [6] to highlight our analysis and derivation of a bound on the tail distribution of the LSA statistic. Specifically we calculate the LSA statistic for a pair of time series, **X **and **Y**. Two-dimensional arrays *P*_*i, j *_and *N*_*i, j *_are used to store the positive and negative partial sums (truncated if less than 0) of the pairwise product of time series values. We also assume a suitable bound on the maximum time lag considered while computing the LSA statistic, denoted by *D*.

The algorithm first initializes the arrays *P*_*j*,0_, *N*_*j*, 0_, *P*_0, *i*_, and *N*_0,*i *_for all *i, j *= 1, ..., *n*, with a maximum absolute difference of *D*. Next it considers the time series pairs for each possible lag, up to a maximum of *D*, and then computes the progressive sum of the pair-wise products of the time-series values from the low to high index of the arrays. During the computation, the progression of the partial sum is reset to 0 if the sum is below 0. After partial sums have been computed, the values of $\hat{N}$ and $\hat{P}$ are calculated by taking the maximum of the corresponding values of the arrays *N *and *P*. Finally, the LSA statistic is estimated as $sign\left(\hat{P}-\hat{N}\right)\frac{\text{max}\left\{\hat{P},\hat{N}\right\}}{n}$.

### Calculating the upper bound

In this section we derive the asymptotic upper bound on the *p*-value for the cumulative probability distribution of the LSA statistic without the need of a normality assumption. Our derivation is based on distributional results of the maximum cumulative sum of independent random variables known in the literature from probability theory [12-15]. We begin by stating our assumptions about the dataset, isolate target calculations from the LSA algorithm, and from our referenced mathematical results, derive and prove important lemmas. These lemmas will serve as the building blocks as we logically construct a theorem which will form the basis of our LSA *p*-value upper bound.

We begin by making certain assumptions about the probability model used to derive the bounds. First, each *P*_*i, j *_or *N*_*i, j *_is considered individually. We assume that the time series values *X_i_, Y_j _*for *i, j *= 1, ..., *n *are *independent *of one another. This assumption can be made when weak dependence exists because it is near the truth and effective, much like the Naive Bayes assumption. This assumption is also enabling, as it allows us to invoke the distributions of partial sums of independent random variables and continue in a mathematically straightforward way. Further, we assume independence between each time time series as a null hypothesis, and as it is subject to rejection upon obtaining a statistically significant LSA value.

Consider lines 5 and 7 of the LSA algorithm (Figure 2), *P*_*i*+*k*+1,*j*+*k*+1 _← max{0, *P*_*i*+*k, j*+*k *_+ *X*_*i*+k _* *Y*_*j*+*k*_} and $\hat{P}\leftarrow {\text{max}}_{\left\{\left(i,j\right):|i-j|\leq D\right\}}\left\{P_{i,j}\right\}$. For any pair of *i *and *j *let us define the sequence random variables as *Z_k _*= *X*_*i*+*k*_*Y*_*j*+*k *_for *k *= 0, ..., min{*n *- *i, n *- *j*} - 1, and the sequence of random variables *ζ_k _*= *Z*_1 _+ ... + *Z_k _*for *k *= 0, ..., min{*n *- *i, n *- *j*} - 1 supposing *ζ*_0 _= 0. Using the above *ζ_k_*'s, we define random variables $\eta_{k}^{*}$ as $\eta_{k}^{*}=\text{max}\left\{\zeta_{1},\zeta_{2},\cdots \;,\zeta_{k}\right\}$ for the same values of *k *= 0, ..., min{*n *- *i, n *- *j*} - 1.

We also define the set of random variables *η*_1_, *η*_2_, ..., *η_k _*by the recurrence formula *η*_*k*+1 _= max{0, *η*_*k *_+ *Z*_*k*+1_}. Note that the random variables *P*_*i*+*k, j*+*k *_and the *η_k _*have the same distribution. It is shown in [12,13] that the random variables $\eta_{k}^{*}$ and *η_k _*also have the same distribution. As a result, now we can analyze the cumulative distribution of *P*_*i*+*k, j*+*k *_as a distribution for $\eta_{k}^{*}$, and use the results by Nevzorov and Petrov [14] on *P*_*i*+*k, j*+*k *_to derive tail probability bounds. We also assume that the random variables *Z_k _*have the first two moments, although such assumptions are not required for the results of [14], we use them to derive simpler bounds.

We now consider a few useful lemmas that we will use to construct our *p*-value upper bound. The first step is to simplify the tail event (which we will later connect to *p*-value) into simpler terms. The following lemma expresses the tail event for LSA {|LSA| >*x*} and any x∈ℝ in terms of the tail events of {*P*_*i, j *_>*x*} and {*N*_*i, j *_>*x*}, the positive and negative LSA calculations for the same *x*, the bound on our test statistic (the target *p*-value).

**Lemma 1 ***For any *x∈ℝ*we have *{|*LSA*| >*x*} = {(∪*_ij_*{*P_ij _*>*xn*}) ∪ (∪*_ij_*{*N_ij _*>*xn*})}.

**Proof**. The result is clear from the following:

$\begin{array}{c} \left\{|LSA|>x\right\}=\left\{max\left\{\hat{P},\hat{N}\right\}>xn\right\}={\left\{\hat{P}\leq xn\cap \hat{N}\leq xn\right\}}^{c}={\left\{max_{ij}\left\{P_{ij}\right\}\leq xn\cap max_{ij}\left\{N_{ij}\right\}\leq xn\right\}}^{c}=\\ {\left\{\left(\cap_{ij}\left\{P_{ij}\leq xn\right\}\right)\cap \left(\cap_{ij}\left\{N_{ij}\leq xn\right\}\right)\right\}}^{c}=\left\{\left(\cup_{ij}\left\{P_{ij}>xn\right\}\right)\cup \left(\cup_{ij}\left\{N_{ij}>xn\right\}\right)\right\} \end{array}$ □

In the LSA algorithm, we have maximums *P_ij _*= *max*{0, *P*_*i*-1, *j*-1 _+ *X*_*i*-1_*Y*_*j*-1_} and *N*_*ij *_= *max*{0, *P*_*i*-1,*j*-1 _- *X*_*i*-1, *j*-1_}, which complicates their theoretical analysis. Fortunately, equivalence have been demonstrated in the literature [12], and we restate these in the following lemma for clarity: the similarity of the distributions of *η_k _*and $\eta_{k}^{*}$, for *k *= 1, ..., min{*n *- *i, n *- *j*} - 1. This will help us derive the bounds for the events {*P_ij _*>*xn*} and {*N_ij _*>*xn*}, the simpler terms we derived in the previous lemma.

**Lemma 2 ***Let Z_i _be mutually independent random variables and let us denote by *$S_{k}=\sum_{i=1}^{k}Z_{i}$*where S*_0 _= 0, *and q*_*k*+1 _= *max*{0, *q_k _*+ *Z_k_*} *with q*_0 _= 0, *then P*(*q_k _*≤ *x*) = *P *(*max*{*S*_0_, ..., *S*_*k*-1_} ≤ *x*) *for *x∈ℝ.

In order to get a simple formula for the bound on the cumulative tail probabilities for *P_i, j _*and *N_i, j _*we reproduce below the results on partial sums of random variables due to Nevzorov and Petrov [14]. For our sequence of *independent and identically distributed *(*iid*) random variables under consideration {*X_n_*} it follows that Lindeberg's condition holds [15]. A property showing the variance of a distribution stabilizes as more variables are added, pinning the tails of it down. Thinking about this in terms of time series, as a series gets larger, the upper bound of the distribution becomes more defined and calculable.

Now to build theorems upon which we will derive a formulaic *p*-value bound.

**Theorem 3 ***If the random variables *{*X_n_*} *have zero expectation and finite variances and if Lindeberg's condition holds: *Λ*_n_*(*ε*) → 0 *as n *→ ∞ ∀*ε *> 0 *where *$\Lambda_{n}\left(\varepsilon \right)=\frac{1}{q_{n}^{2}}\sum_{k=1}^{n}\int_{\left\{|x|>\varepsilon q_{n}\right\}}x^{2}dV_{k}\left(x\right)$*and *$q_{n}^{2}=\sum_{k=1}^{n}E\left(X_{k}^{2}\right)$*and *$G\left(x\right)=\sqrt{\frac{2}{\pi }}\int_{0}^{x}e^{-t^{2}/2}dt$*if x *≥ 0 *and *0 *if x *< 0*, then we have *${\text{sup}}_{x}|P\left({\bar{S}}_{n}<q_{n}x\right)-G\left(x\right)|\to 0$*where *${\bar{S}}_{n}={\text{max}}_{1\leq k\leq n}\sum_{j=1}^{k}X_{j}$*and V_k_*(*x*) = **P**(*X_k _*≤ *x*)

In order to apply the above theorem to get a simple formulaic approximation, we assume some random variables ${\left\{Z_{i}\right\}}_{1}^{m}$, each with the variance *σ*^2 ^and $S_{k}=\sum_{i=1}^{k}Z_{i}$. Then by applying the above theorem, we get the following uniform convergence of distribution to that of the one-sided standard normal as ${\text{sup}}_{x}|P\left(max_{k\in \left\{1,\ldots ,m\right\}}S_{k}\leq \sqrt{m}\sigma x\right)-G\left(x\right)|\;\to 0$*as m *→ ∞.

Now we use the above results to get the probability estimates for our simple event terms {*P_ij _*>*xn*} and {*N_ij _*>*xn*}. The following theorem provides us with the *p*-value's tail bound for LSA for any x∈ℝ.

**Theorem 4 ***For G, the one-sided normal distribution, defined above *$\text{P}\left(|LSA|>x\right)\leq 2\left(n^{2}-\left(n-D-1\right)\left(n-D\right)\right)\left(1-G\left(x\sqrt{n/V\alpha r\left(X_{1}Y_{1}\right)}\right)\right).$

**Proof**. By applying Lemma 2 we have

$$\begin{array}{c} P\left(P_{ij}>xn\right)=P\left(\text{max}\left\{0,\;P_{i-1,j-1}+X_{i-1}Y_{j-1}\right\}>xn\right)\\ =1-P\left(\text{max}\left\{0,\;P_{i-1,j-1}+X_{i-1}Y_{j-1}\right\}\leq xn\right)\\ =1-P\left(\underset{1\leq k\leq \text{min}\left\{i-1,j-1\right\}}{\text{max}}\left\{\overset{k}{\underset{l=0}{\sum }}X_{l}Y_{l}\right\}\leq xn\right), \end{array}$$

and by Theorem 3, replacing *x *with *y*, we have

$$\underset{y}{\text{sup}}|P\left(\underset{k\in \left\{1,\ldots ,m\right\}}{\text{max}}S_{k}\leq \sqrt{m}\sigma y\right)-G\left(y\right)|\;\to 0\;as\;m\to \infty .$$

Notice that $\sum_{l=0}^{k}X_{l}Y_{l}$ satisfies the definition of *S_k_*, so replacing *S_k_*, $\sqrt{m}\;$, and *σ *with $\sum_{l=0}^{k}X_{l}Y_{l}$, $\sqrt{\text{min}\left\{i-1,j-1\right\}}$, and $\sqrt{Var\left(X_{1}Y_{1}\right)}$, respectively,

$$\underset{y}{\text{sup}}\left|P\left({\text{max}}_{k\in \left\{1,\ldots ,\sqrt{\text{min}\left\{i-1,j-1\right\}}\right\}}\overset{k}{\underset{l=0}{\sum }}X_{l}Y_{l}\leq \sqrt{\text{min}\left\{i-1,j-1\right\}Var\left(X_{1}Y_{1}\right)}y\right)-G\left(y\right)\right|\to 0,$$

*as n *→ ∞, and by change of variables to get our equation into the appropriate form

$$\begin{array}{c} xn=\sqrt{\text{min}\left\{i-1,j-1\right\}Var\left(X_{1}Y_{1}\right)}y\Rightarrow y=xn/\sqrt{\text{min}\left\{i-1,j-1\right\}Var\left(X_{1}Y_{1}\right)}\\ \Rightarrow \underset{x}{\text{sup}}\left|P\left({\text{max}}_{k\varepsilon \left\{1,\ldots ,\sqrt{\text{min}\left\{i-1,j-1\right\}}\right\}}\overset{k}{\underset{l=0}{\sum }}X_{l}Y_{l}\leq xn\right)-G\left(xn/\sqrt{\text{min}\left\{i-1,j-1\right\}Var\left(X_{1}Y_{1}\right)}\right)\right|\to 0, \end{array}$$

*as n *→ ∞, thus

$$P\left(P_{ij}>xn\right)≅1-G\left(xn/\sqrt{min\left\{i-1,j-1\right\}Var\left(X_{1}Y_{1}\right)}\right).$$

It follows from Boole's inequality and Lemma 1 that

$$\begin{array}{c} P\left(|LSA|\;>x\right)=P\left(\left(\cup_{ij}\left\{P_{ij}>xn\right\}\right)\cup \left(\cup_{ij}\left\{N_{ij}>xn\right\}\right)\right)\\ \leq \underset{ij}{\sum }P\left(P_{ij}>xn\right)+\underset{ij}{\sum }P\left(N_{ij}>xn\right)\\ =2\underset{ij}{\sum }\left(1-G\left(xn/\sqrt{min\left\{i-1,j-1\right\}Var\left(X_{1}Y_{1}\right)}\right)\right). \end{array}$$

Finally, we have the following tail probability bound

$$\begin{array}{c} \text{P}\left(|LSA|\;>x\right)\leq 2\underset{ij}{\sum }\left(1-G\left(xn/\sqrt{min\left\{i-1,j-1\right\}Var\left(X_{1}Y_{1}\right)}\right)\right)\\ \leq 2\underset{ij}{\sum }\left(1-G\left(xn/\sqrt{nVar\left(X_{1}Y_{1}\right)}\right)\right)=2\left(n^{2}-\left(n-D-1\right)\left(n-D\right)\right)\left(1-G\left(x\sqrt{n/Var\left(X_{1}Y_{1}\right)}\right)\right), \end{array}$$

standardizing with a mean of zero and a variance of one

$$=2\left(n^{2}-\left(n-D-1\right)\left(n-D\right)\right)\left(1-G\left(x\sqrt{n}\right)\right).$$

□

**Figure 3:**
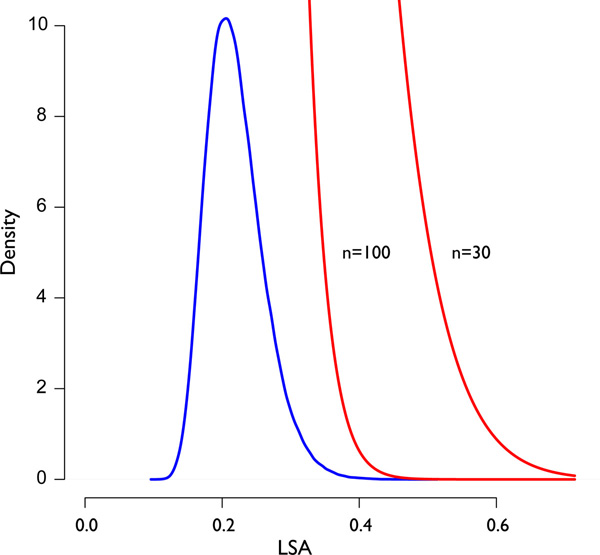
**Asymptotic *p*-value upper bounds converge on the LSA density**. Notice that the *p*-value upper bound (red) converges in the tail to the approximate LSA density (blue), an attractive quality. As the number of time steps (*n*) increase, both the density and the *p*-value upper bound push up against zero. This is similar to the asymptotic behaviour of PCC.

Note that this last result is asymptotic. Thus, *n *must be substantially large for this *p*-value bound to be relevant (Figure 3 and Table 1). Similar to the normal distribution as an approximation to Student's *t*-distribution, this implementation of LSA requires at least 30 time points to promote confidence. Though this convergence can vary from dataset to dataset, the bound is conservative, and will not easily produce false positives if run on shorter time series.

**Table 1:** Empirical *p*-value (Emp) & the FASTLSA *p*-value bound (Fas) with n = 30, 50, & 100 time steps.

x1	n30Emp	n30Fas	n50Emp	n50Fas	n100Emp	n100Fas
0.05	1	1.000	1	1.000	1	1.000
0.07	1	1.000	1	1.000	0.997	1.000
0.09	1	1.000	0.999	1.000	0.953	1.000
0.11	0.999	1.000	0.984	1.000	0.819	1.000
0.13	0.989	1.000	0.928	1.000	0.627	1.000
0.15	0.958	1.000	0.823	1.000	0.441	1.000
0.17	0.896	1.000	0.687	1.000	0.292	1.000
0.19	0.803	1.000	0.545	1.000	0.184	1.000
0.21	0.694	1.000	0.417	1.000	0.111	1.000
0.23	0.58	1.000	0.309	1.000	0.064	1.000
0.25	0.472	1.000	0.224	1.000	0.036	1.000
0.27	0.376	1.000	0.158	1.000	0.019	0.693
0.29	0.294	1.000	0.109	1.000	0.009	0.373
0.31	0.227	1.000	0.073	1.000	0.005	0.194
0.33	0.172	1.000	0.048	0.981	0.002	0.097
0.35	0.128	1.000	0.031	0.666	0.001	0.047
0.37	0.094	1.000	0.019	0.444	< 0.001	0.022
0.39	0.067	0.98	0.012	0.291	< 0.001	0.01
0.41	0.048	0.742	0.007	0.187	< 0.001	0.004
0.43	0.033	0.555	0.004	0.118	< 0.001	0.002
0.45	0.023	0.411	0.002	0.073	< 0.001	0.001
0.47	0.015	0.301	0.001	0.044	< 0.001	< 0.001
0.49	0.01	0.218	0.001	0.027	< 0.001	< 0.001
0.51	0.006	0.156	< 0.001	0.016	< 0.001	< 0.001
0.53	0.004	0.111	< 0.001	0.009	< 0.001	< 0.001
0.55	0.002	0.078	< 0.001	0.005	< 0.001	< 0.001
0.57	0.001	0.054	< 0.001	0.003	< 0.001	< 0.001
0.59	0.001	0.037	< 0.001	0.002	< 0.001	< 0.001
0.61	< 0.001	0.025	< 0.001	0.001	< 0.001	< 0.001
0.63	< 0.001	0.017	< 0.001	< 0.001	< 0.001	< 0.001
0.65	< 0.001	0.011	< 0.001	< 0.001	< 0.001	< 0.001
0.67	< 0.001	0.007	< 0.001	< 0.001	< 0.001	< 0.001
0.69	< 0.001	0.005	< 0.001	< 0.001	< 0.001	< 0.001
0.71	< 0.001	0.003	< 0.001	< 0.001	< 0.001	< 0.001
0.73	< 0.001	0.002	< 0.001	< 0.001	< 0.001	< 0.001
0.75	< 0.001	0.001	< 0.001	< 0.001	< 0.001	< 0.001
0.77	< 0.001	0.001	< 0.001	< 0.001	< 0.001	< 0.001
0.79	< 0.001	< 0.001	< 0.001	< 0.001	< 0.001	< 0.001
0.81	< 0.001	< 0.001	< 0.001	< 0.001	< 0.001	< 0.001
0.83	< 0.001	< 0.001	< 0.001	< 0.001	< 0.001	< 0.001
0.85	< 0.001	< 0.001	< 0.001	< 0.001	< 0.001	< 0.001
0.87	< 0.001	< 0.001	< 0.001	< 0.001	< 0.001	< 0.001
0.89	< 0.001	< 0.001	< 0.001	< 0.001	< 0.001	< 0.001
0.91	< 0.001	< 0.001	< 0.001	< 0.001	< 0.001	< 0.001
0.93	< 0.001	< 0.001	< 0.001	< 0.001	< 0.001	< 0.001
0.95	< 0.001	< 0.001	< 0.001	< 0.001	< 0.001	< 0.001
0.97	< 0.001	< 0.001	< 0.001	< 0.001	< 0.001	< 0.001
0.99	< 0.001	< 0.001	< 0.001	< 0.001	< 0.001	0.001

## Results

To validate versatility and effectiveness of the derived upper bound (Theorem 4), we applied the algorithm to four datasets, two sourced from biology, one from social networking, and a randomly generated control dataset. These include the Moving Pictures of the Human Microbiome [8] (MPH), the largest human microbial time series to date, a microarray hybridization dataset identifying cell cycle regulated genes in the yeast *Saccharomyces cerevisiae *[9] (CDC), and an online social media dataset of the volumes of the top 1000 Memetracker phrases and top 1000 twitter hash tags over an eight month period from September 2008 to August 2009 [10]. Missing data values were interpolated by averaging the two nearest temporal data points, and all analysis was performed on a Mac Pro desktop computer running Mac OSX 10.6.8 with a 2 × 2.4 Ghz Quad-Core Intel Xeon processors and 16 GB of 1066 Mhz DDR3 RAM.

### Computational complexity

**Figure 4:**
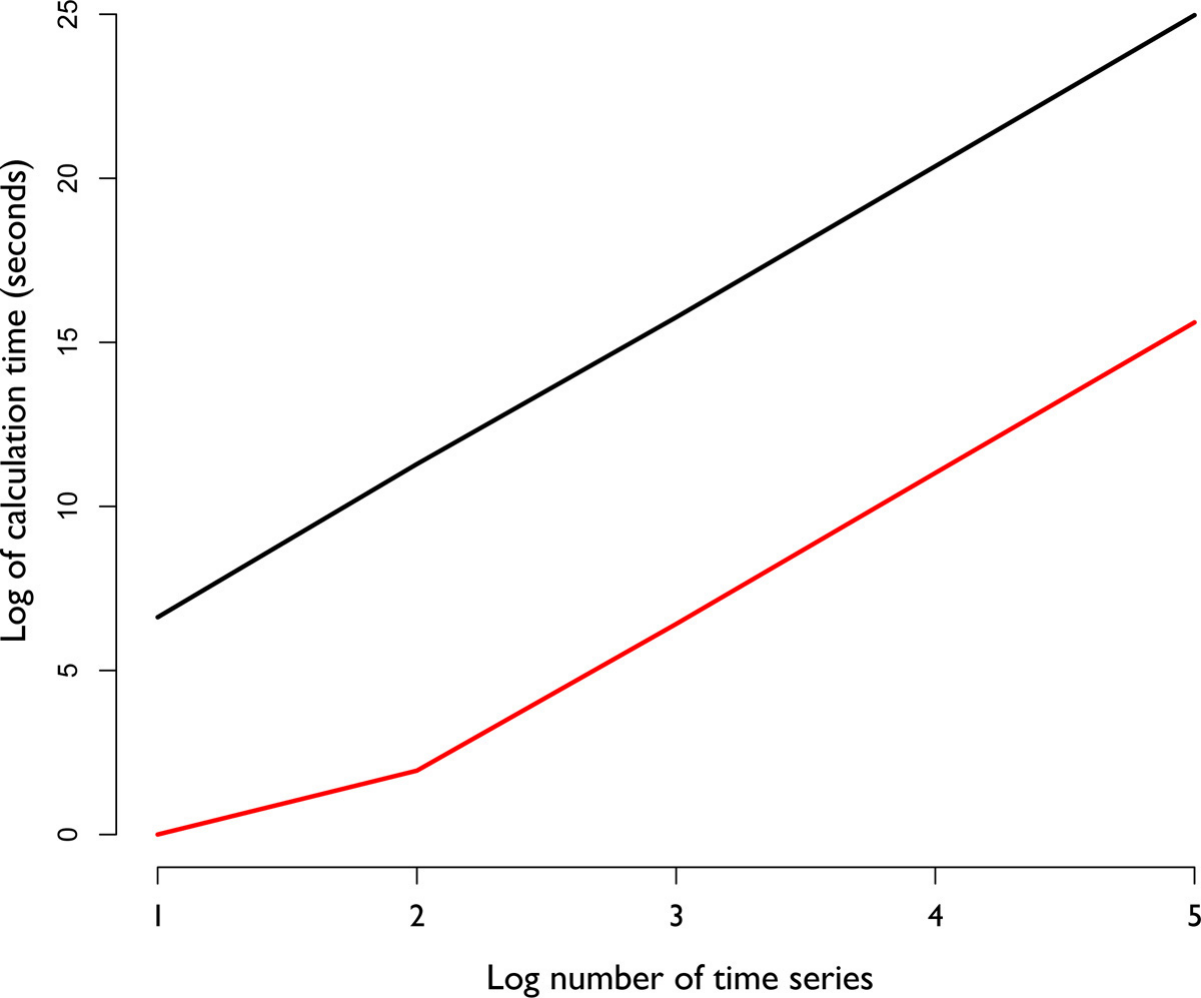
**LSA calculation time as a function of the number of time series**. On this log-log plot notice that because of its lack of a permutation test FASTLSA (red) is consistently faster than the older implementation of LSA (black) [6].

The algorithm calculates in *O*(*m*^2^*n*) time, where *m *is the number of time series and *n *is the length of each time series. To get an idea of how long calculations take, we fixed *n *= 50, *d *= 3 and plotted log-calculation time against log-*m *(Figure 4). It can be seen that LSA tests with *p*-values calculated by the permutation test are about 10,000 times slower than calculating *p*-values formulaically. Compared to direct formulaic calculation, random number generation is slow, making a repetition of 10,000 permutations for each time series pair a computationally intense operation (Table 2). The permutation test may be able to calculate statistically significant (*α *= 0.001) pairs confidently, but applying a multiple test correction (Bonferroni) will require exponentially more permutations to reach the same level of confidence for the entire dataset. Pairwise comparisons for big datasets are computationally infeasible to sufficiently estimate *p*-values with enough accuracy to protect against false positives. In contrast, FASTLSA directly calculates a conservative upper bound approximating the *p*-value, making permutation unnecessary and protecting against false positives.

**Table 2:** Empirical running time for LSA calculation for data sets of different size

	Time series	Time points	fastLSA (single thread)	fastLSA (16 threads)
Twitter	1,000	130	6 sec	1 sec
CDC	6,178	24	3.24 min	2.2 sec
MPH	14,105	390	58 min	7.5 min
First Null	100,000	100	-	54 min
Second Null	1,000,000	30	-	2 days 3 hrs
Third Null	1,000,000	100	-	7 days 23 hrs

### Moving pictures of the human microbiome (MPH)

The MPH time series dataset [8] investigates temporal variations in the microbial community structure of two healthy human subjects, one male, one female. Samples were collected from three body parts, the gut (feces), mouth, and skin (left and right palms) daily for 15 months (male) and six months (female) with taxonomy being determined by the amplified V4 region of the small subunit ribosomal RNA (SSU or 16S rRNA) gene. The male and female samples were concatenated together resulting in a profile of 14105 taxa for 390 time points with missing values being interpolated by the average of the two nearest time points.

**Figure 5:**
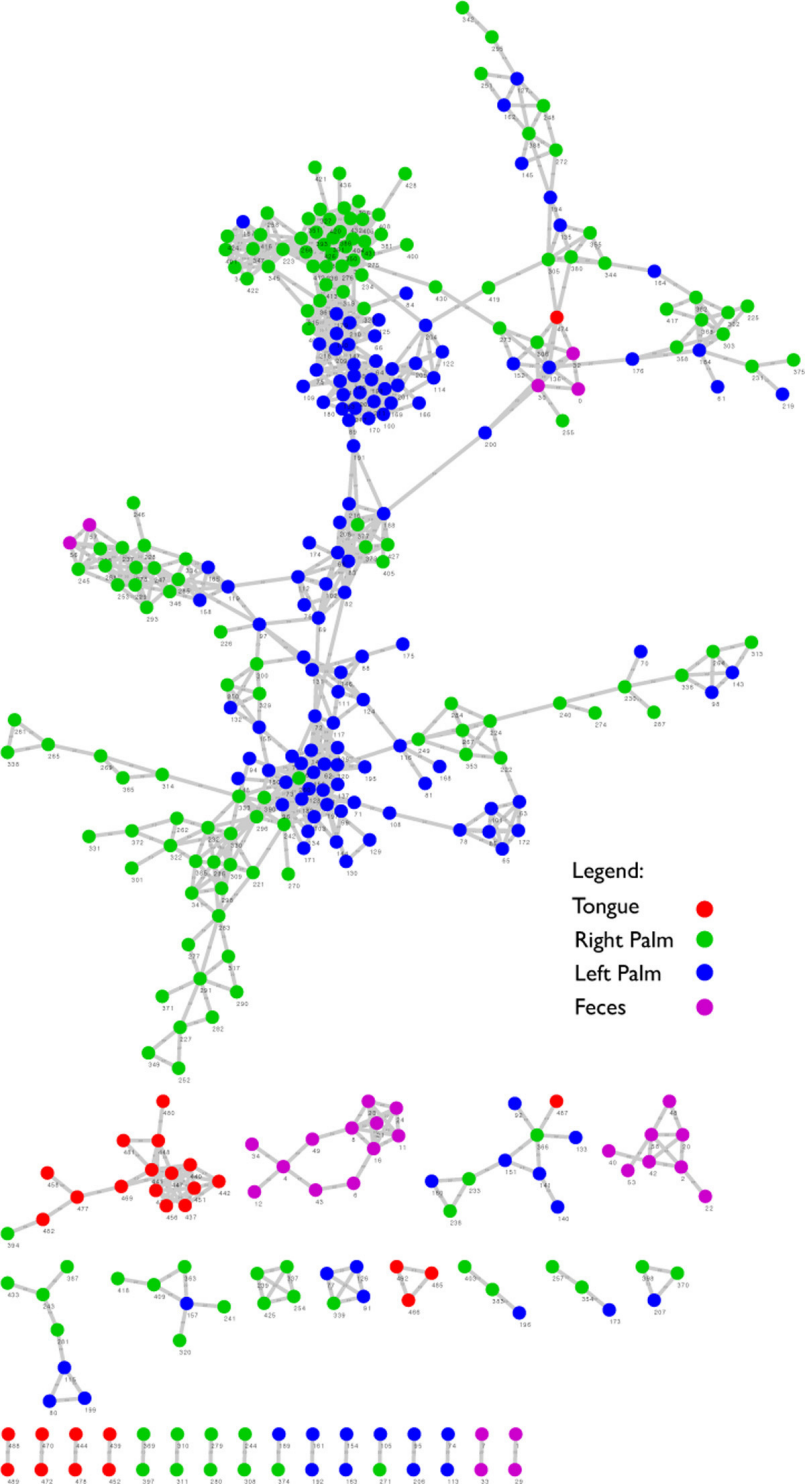
**MPH local similarity graph**. A local similarity graph of the MPH dataset showing significant LSA values as defined by the asymptotic upper bound (*α *= 0.001). Local clusters defined by LSA were revealed and the mapping of samples sources (feces, mouth, and skin) to the nodes revealed underlying network structure.

For a given time series, if more than 25% of time steps were zero it was removed from the analysis. Analysis took 58 minutes (7.5 minutes on 16 threads) without including output writing time which is variable. Significant (*α *< 0.001) LSA results revealed clusters of local similarity that corresponded well when nodes were colored by sample source (Figure 5). The low level of mixing between local clusters reflects the large differences in taxonomic profiles across the different body environments [8].

### Microarray hybridization detection cell cycle-regulated genes in yeast *Saccharomyces cerevisae *(CDC)

**Figure 6:**
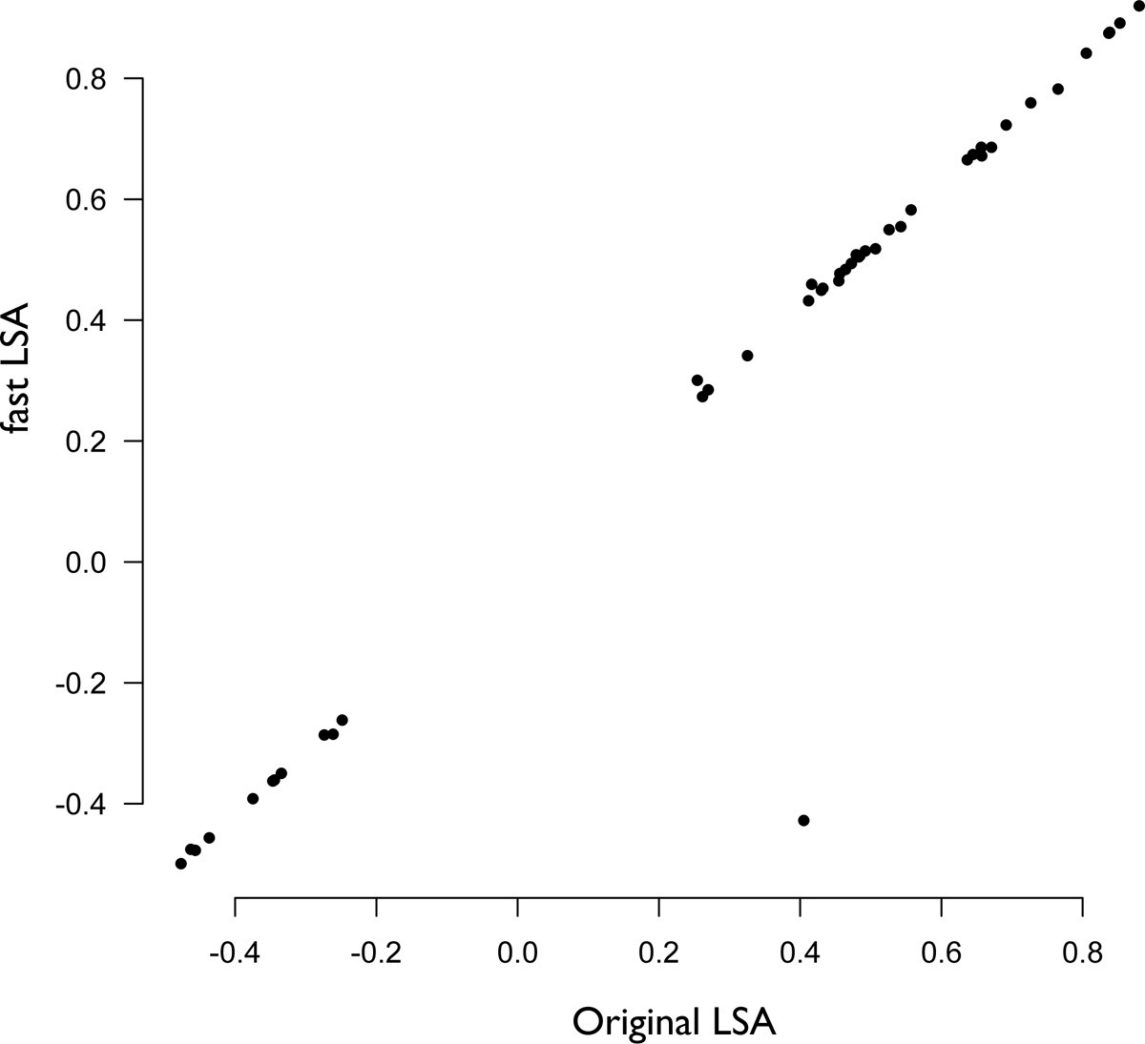
**Comparison of LSA values: fastLSA and Original LSA**. A comparison of calculated LSA statistics between FASTLSA and Original LSA implemented by [6] and calculated on the CDC dataset. There is an almost one-to-one correspondence between calculated values. The one outlying value was likely due to the transform that the original LSA applies, causing a disagreement between positive and negative values. For a single value fastLSA picked the negative value (-0.4) and original LSA picked positive (0.4).

In the CDC data set [9], we focused on the *cdc*15 temperature sensitive mutant and the profile of 6178 genes over 24 time steps, representing gene expression for approximately three cell cycles. Analysis took 3.25 minutes (22 seconds on 16 threads) without including output writing time (Figure 6). Applying the asymptotic bound with the small number of time steps resulted in some rather large bounds (≥ 1).

**Figure 7:**
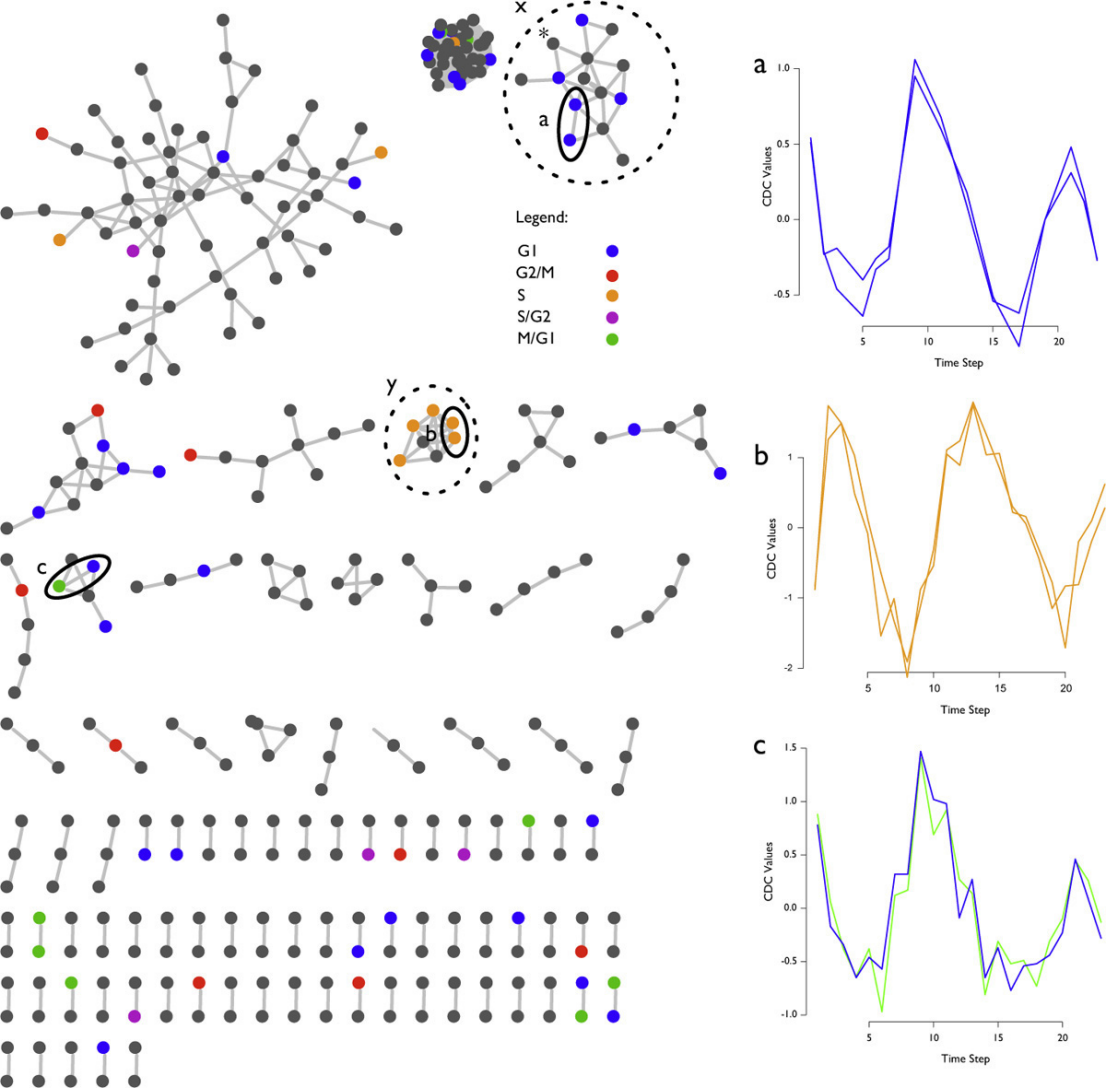
**CDC local similarity graph**. A local similarity graph of the CDC dataset showing significant LSA values as defined by the upper bound cutoff and the additional constraint of absolute LSA values greater than 0.85 (*α *= 0.001, |*LSA*| ≥ 0.85). Clusters of local structure are observed with some example correlates of expression shown in graphs a, b, and c, indicated by solid ellipses. However, because only 800 of the approximately 6,000 genes could be classified to a cell cycle position (G1, G2/M, S, S/G2, M/G1) we could only guess at two clusters' functional characteristics. Cluster **x **has many G1 genes that, according to the Saccharomyces Genome Database http://www.yeastgenome.org [18], have some functionality relative to helicases and telomeres. The gene, YKR077W (*), accelerates the cell cycle initiation stage (G1) when abundant [19]. Cluster **y **is a set of genes encoding histone proteins [19]. Histone development is an essential part of genome replication [20] adequately describing all of cluster **y **as S phase genes.

However, LSA was capable of detecting lead-lag correlation despite the periodicity of the data, demonstrating its capacity to find long correlate pairs with a large number of covariate time series. Only 800 of the 6178 gene nodes could be classified from [9] to one of the five defined cell cycle phases (G1, G2/M, S, S/G2, M/G1) so only two clusters could be inferred upon with any confidence (Figure 7).

### Social media: top 1000 Twitter and Memetracker phrases (Twitter)

**Figure 8:**
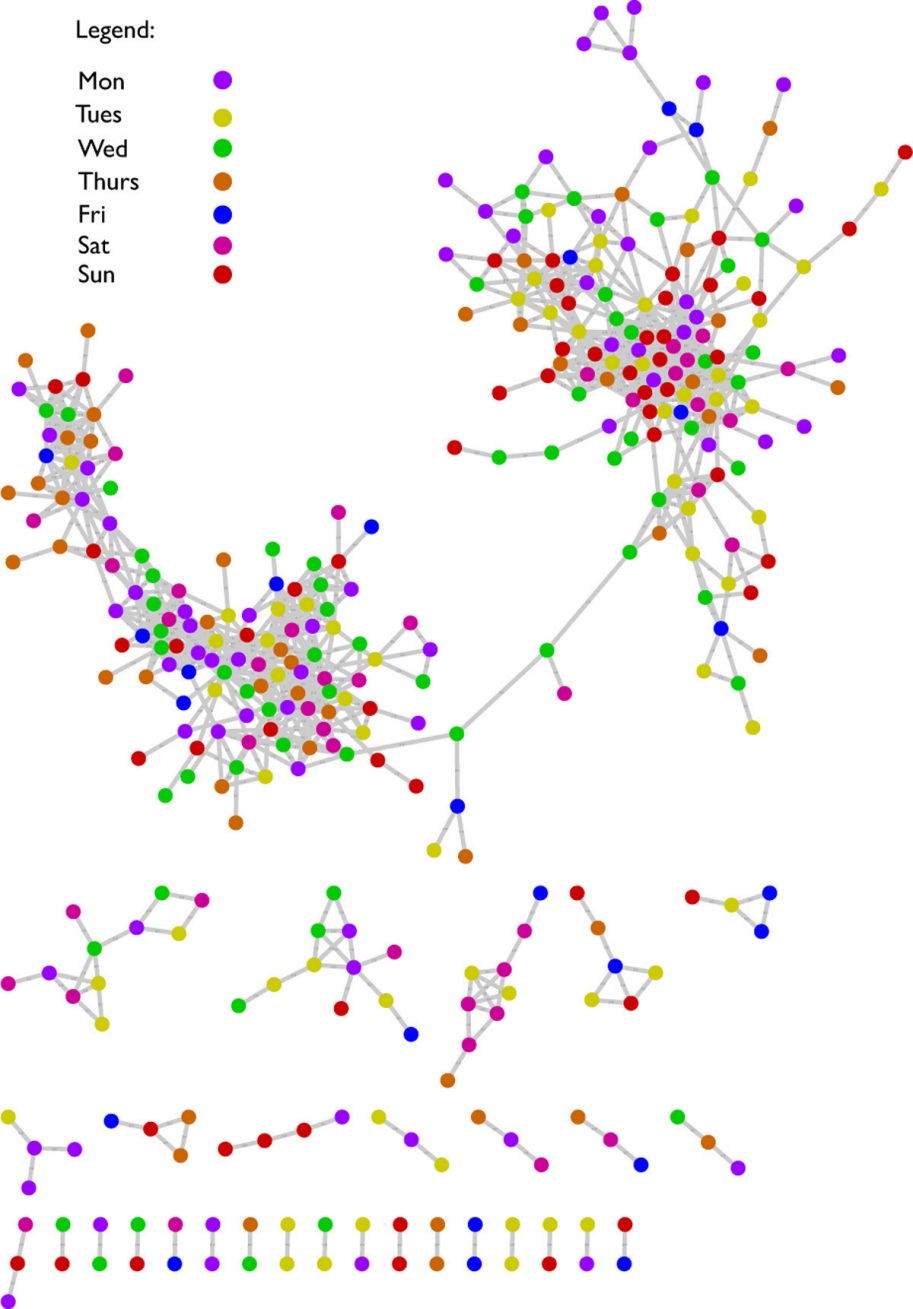
**Twitter local similarity graph**. A local similarity graph of the Twitter dataset showing significant LSA values with an additional threshold absolute LSA values greater than 0.98 (*α *= 0.001,|*LSA *≥ 0.98). Two primary clusters of local similarity were found, however, none of the attempted metadata mappings could classify the clusters by time (hour of day, day of week, season, etc.) or general message content (political, personal, media, etc.).

The data from [10] contains the volume of the top 1000 Twitter and Memetracker phrase counts over 130 time steps from September 2008 to August 2009, a spacing of approximately 2-3 days per observation. Analysis took approximately six seconds (one second on 16 threads) without including write out time. Two major clusters of related times series nodes emerged. However, attempts to label the series using existing metadata of general content or time granularity (day of the week, working hours, seasonality, etc.) did not elucidate its structure (Figure 8). We conjecture that this difference is geographical (East-West North America) or socially structured, however, additional metadata on geolocation or social connectivity associations of the nodes would be needed to better elucidate network structure.

### Null hypothesis simulated data

**Figure 9:**
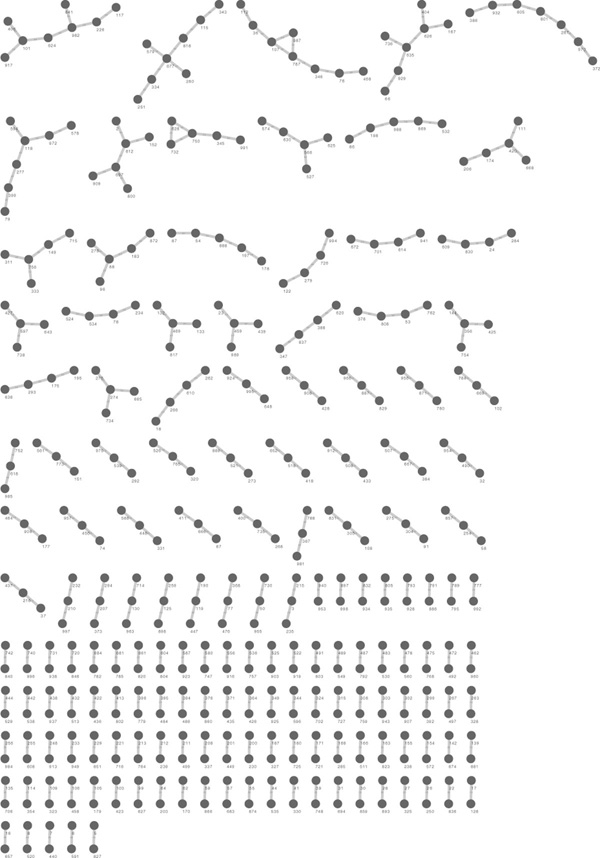
**Uniform random local similarity graph**. A local similarity graph representing purposeful false positives, 1000 time series with 100 time steps randomly generated from a uniform distribution. Notice how no cliques form in the random data generated from a uniform distribution.

Finally, to identify throughput limits of FASTLSA and to simulate a large *iid *dataset without time dependence, three data matrices were randomly generated: (1) one hundred thousand measurements across 100 time steps, (2) one million measurements across 30 time steps, and (3) one million measurements across 100 time steps. Data were generated by random sampling from a uniform distribution. Running FASTLSA on 16 threads, the first dataset (100, 000 × 100) took one hour 54 minutes, the second (1, 000, 000 × 30) 2 days and 3 hours, and the third (1, 000, 000 × 100) had an ETA of 7 days and 23 hours without including writeout time. The asymptotic bound is conservative for shorter datasets (*n *≤ 30) (Figure 3, Table 1), the second data having 30 time points found zero false positives, despite having a Bonferroni correction of $\alpha /)\left(\begin{array}{c} n\\ 2 \end{array}\right)=10^{-13}$. This is likely because the software's *p*-value is an upper bound to the real *p*-value, and so is the Bonferroni correction. An inspection of a uniform random graph (*α *= 0.05, |*LSA*| < 0.4) of 1,000 random time series with 100 time steps did not generate any cliques, but only short (4-8) length chains of nodes, serving as a warning to those wanting to interpret relevant structure (Figure 9). Given appropriate thresholds on LSA values, cliques do not seem to occur randomly.

## Discussion

FASTLSA uses a novel asymptotic upper bound algorithm for calculating the LSA *p*-value. This is done without any normality assumption, extending implementation to untransformed data and data in violation of normality assumptions such as time series containing many zero entries. Moreover, FASTLSA replaces a computationally intensive permutation test that was previously required to calculate significance of LSA statistics with a dramatic increase on the size of datasets that can be analyzed on a single desktop machine. However, like all asymptotic bounds, a significant number of observations need to be obtained for their application. From theoretical simulation, we estimate this to be greater than 30 time points for most datasets. This is supported by our experience on the CDC and MPH datasets having 24 and 390 time series, respectively. Despite this potential operating constraint, FASTLSA expands the boundaries of LSA allowing time series analysis on datasets with millions of co-variate time series. The algorithm is implemented as a computationally efficient software package, FASTLSA, written in C and optimized for threading on multi-core computers using POSIX threads. Finally, we demonstrated the utility and versatility of FASTLSA using real-world and simulated time series datasets from different fields of inquiry, visualizing the resulting clusters of local similarity using the Cytoscape software.

LSA statistics have been demonstrated to capture relevant local similarity structure for a number of biological datasets [16,17]. However, previous implementations were limited to relatively small datasets. FASTLSA improves the computational efficiency and statistical robustness of LSA, a necessary step in using the statistic to explore next generation time series datasets. Despite the current improvements, the structure captured by LSA is less than ideal and could be further improved. Given two vectors of time series, LSA reports the strongest statistic. However, it is unclear where this significant time window occurs, or if there are multiple small windows with large LSA values that are not reported. An inspection of time series traces in question is often required to visually check exactly how the two are similar. Another hazard is that LSA does not handle missing data effectively, and so a continuous version of the statistic would be desirable for exploratory experiments where sampling conditions could change to small degrees and analysis could be performed without imputation. Furthermore, LSA is asymmetric in nature, meaning that time reversal has the potential to produce differing LSA values. We anticipate even better performance from the statistic if these issues were addressed, perhaps through a modified version of PCC that isolates optimal windows of similarity.

## Conclusions

LSA is a local similarity statistic that has recently been used to capture relevant local structure in time series datasets, particularly within the biological community. However, its use has been limited to smaller datasets due to an intensive permutation test used to calculate significance. Our derivation and direct calculation of an asymptotic upper bound using FASTLSA replaces this onerous calculation without a normality assumption, enabling LSA on time series datasets containing millions of co-variate time series. We demonstrate the utility and versatility of FASTLSA by analyzing time series data from public health, microbial ecology, and social media and compare these results to the previous implementation of LSA, obtaining similar results with orders of magnitude increase in throughput.

**Project name: **fastLSA

**Project home page: **http://www.cmde.science.ubc.ca/hallam/fastLSA/

**Operating system(s): **OS X, Linux, or Windows

**Programming Languages: **C /C++

**Other requirements: **1 GB RAM

**License: **GPLv3

**Non-academic restrictions: **None

## List of abbreviations

**LSA: **Local Similarity Analysis; **PCC: **Pearson's Correlation Coefficient; **PCA: **Principal Component Analysis; **MDS: **Multidimensional Scaling; **DFA: **Discriminant Fraction Analysis; **MPH: **Moving Pictures of the Human Microbiome; **CDC: **Centre of Disease Control.

## Competing interests

The authors declare that they have no competing interests.

## Authors' contributions

WED derived the *p*-value upper bound and was the primary programmer of the LSA statistics. NH assisted in the derivation of the upper bound, the analysis of the MPH, CDC, and Twitter datasets, and was the primary manuscript writer and editor. KK validated upper bound result and assisted in the multi-threaded implementation of the software. SH oversaw the research and managed the group.

## Declarations

The publication costs for this article were funded by Genome British Columbia and Genome Canada.

This article has been published as part of *BMC Genomics* Volume 14 Supplement 1, 2013: Selected articles from the Eleventh Asia Pacific Bioinformatics Conference (APBC 2013): Genomics. The full contents of the supplement are available online at http://www.biomedcentral.com/bmcgenomics/supplements/14/S1.

## References

[B1] Lynch C Big data: How do your data grow? Nature 2008 455 7209 28 29 10.1038/455028a 18769419

[B2] Bell G Hey T Szalay A Computer science. Beyond the data deluge Science 2009 323 5919 1297 1298 10.1126/science.1170411 19265007

[B3] Schadt EE Linderman MD Sorenson J Lee L Nolan GP Computational solutions to large-scale data management and analysis Nature Reviews Genetics 2010 11 9 647 657 10.1038/nrg2857 PMC3124937 20717155

[B4] Ranjard L Poly F Lata JC Mougel C Thioulouse J Nazaret S Characterization of bacterial and fungal soil communities by automated ribosomal intergenic spacer analysis fingerprints: biological and methodological variability Applied and Environmental Microbiology 2001 67 10 4479 4487 10.1128/AEM.67.10.4479-4487.2001 11571146 PMC93193

[B5] Mooy BASV Devol AH Keil RG Relationship between bacterial community structure, light, and carbon cycling in the eastern subarctic North Pacific Limnology and Oceanography 2004 1056 1062

[B6] Ruan Q Dutta D Schwalbach MS Steele JA Fuhrman JA Sun F Local similarity analysis reveals unique associations among marine bacterioplankton species and environmental factors Bioinformatics 2006 22 20 2532 2538 10.1093/bioinformatics/btl417 16882654

[B7] Xia LC Steele JA Cram JA Cardon ZG Simmons SL Vallino JJ Fuhrman JA Sun F Extended local similarity analysis (eLSA) of microbial community and other time series data with replicates BMC Syst Biol 2011 5 Suppl 2 S15 10.1186/1752-0509-5-S2-S15 22784572 PMC3287481

[B8] Caporaso JG Lauber CL Costello EK Berg-Lyons D Gonzalez A Stombaugh J Knights D Gajer P Ravel J Fierer N Gordon JI Knight R Moving pictures of the human microbiome Genome Biol 2011 12 R50 10.1186/gb-2011-12-5-r50 21624126 PMC3271711

[B9] Spellman PT Sherlock G Zhang MQ Iyer VR Anders K Eisen MB Brown PO Botstein D Futcher B Comprehensive identification of cell cycle-regulated genes of the yeast *Saccharomyces cerevisiae* by microarray hybridization Molecular Biology of the Cell 1998 9 12 3273 3297 9843569 10.1091/mbc.9.12.3273 PMC25624

[B10] Yang J Leskovec J Patterns of temporal variation in online media Proceedings of the Fourth ACM International Conference on Web Search and Data Mining 2011 177 186

[B11] Shannon P Markiel A Ozier O Baliga NS Wang JT Ramage D Amin N Schwikowski B Ideker T Cytoscape: a software environment for integrated models of biomolecular interaction networks Genome Research 2003 13 11 2498 2504 10.1101/gr.1239303 14597658 PMC403769

[B12] Takacs L On the distribution of the maximum of sums of mutually independent and identically distributed random variables Advances in Applied Probability 1970 2 344 354 10.2307/1426323

[B13] Wald A On the distribution of the maximum of successive cumulative sum of independent but not identically distributed chance variables Bulletin of the American Mathematical Society 1948 54 422 430 10.1090/S0002-9904-1948-09021-8

[B14] Nevzorov VB Petrov VV On the distribution of the maximum cumulative sum of independent random variables Theory of Probability and its Applications 1969 14 4 682 687 10.1137/1114083

[B15] Lindeberg J Eine neue Herleitung des Exponentialgesetzes in der Wahrscheinlichkeitsrechnung Mathematische Zeitschrift 1922 15 211 225 10.1007/BF01494395

[B16] Fuhrman JA Steele JA Community structure of marine bacterioplankton: patterns, networks, and relationships to function Aquatic Microbial Ecology 2008 53 69 81

[B17] Steele JA Countway PD Xia L Vigil PD Beman JM Kim DY Chow CET Sachdeva R Jones AC Schwalbach MS Rose JM Hewson I Patel A Sun F Caron DA Fuhrman JA Marine bacterial, archaeal and protistan association networks reveal ecological linkages The ISME Journal 2011 5 9 1414 1425 10.1038/ismej.2011.24 21430787 PMC3160682

[B18] Cherry JM Hong EL Amundsen C Balakrishnan R Binkley G Chan ET Christie KR Costanzo MC Dwight SS Engel SR Fisk DG Hirschman JE Hitz BC Karra K Krieger CJ Miyasato SR Nash RS Park J Skrzypek MS Simison M Weng S Wong ED Saccharomyces Genome Database: the genomics resource of budding yeast Nucleic Acids Res 2012 40 D700 D705 10.1093/nar/gkr1029 22110037 PMC3245034

[B19] Ashe M deBruin RA Kalashnikova T McDonald WJ Yates JR III Wittenberg C The SBF- and MBF-associated protein Msa1 is required for proper timing of G1-specific transcription in *Saccharomyces cerevisae* Journal of Biological Chemistry 2007 283 6040 6049 18160399 10.1074/jbc.M708248200

[B20] Ewen ME Where the cell cycle and histones meet Genes Dev 2000 14 2265 2270 10.1101/gad.842100 10995383

